# Supplementary material for: Channelized Substrates Made from BaZr0.75Ce0.05Y0.2O3−d Proton-Conducting Ceramic Polymer Clay
Source: Membranes (Basel). 2019 Oct 9;9(10):130. doi: 10.3390/membranes9100130 (PMC6836173; doi:10.3390/membranes9100130)
Supplement: Supplementary file 1 [file membranes-09-00130-s001.pdf]

membranes

MDPI

Supplementary Materials: Channelized Substrates Made from BaZr$_{0.75}$Ce$_{0.05}$Y$_{0.2}$O$_{3-d}$ Proton-Conducting Ceramic Polymer Clay

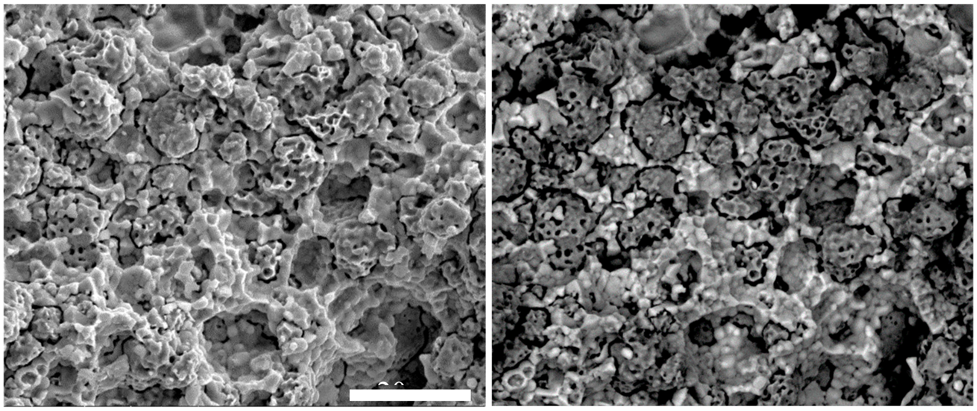

Figure S1. Secondary electron (left) and back-scattered electron (right) micrographs of the BZCY755/Ni electrode in a sintered and reduced BZCY755/Ni//BZCY755//BZCY755/Ni fractured cross section. The sample was reduced at 700 ºC. (BZCY755: light phase, Ni: grey phase).
